# Length of Stay, Hospital Costs and Mortality Associated With Comorbidity According to the Charlson Comorbidity Index in Immobile Patients After Ischemic Stroke in China: A National Study

**DOI:** 10.34172/ijhpm.2021.79

**Published:** 2021-08-07

**Authors:** Hongpeng Liu, Baoyun Song, Jingfen Jin, Yilan Liu, Xianxiu Wen, Shouzhen Cheng, Stephen Nicholas, Elizabeth Maitland, Xinjuan Wu, Dawei Zhu

**Affiliations:** ^1^Department of Nursing, Chinese Academy of Medical Sciences - Peking Union Medical College, Peking Union Medical College Hospital, Beijing, China.; ^2^Department of Nursing, Henan Provincial People’s Hospital, Zhengzhou, China.; ^3^Department of Nursing, The Second Affiliated Hospital Zhejiang University School of Medicine, Hangzhou, China.; ^4^Department of Nursing, Wuhan Union Hospital, Wuhan, China.; ^5^Department of Nursing, Sichuan Provincial People’s Hospital, Chengdu, China.; ^6^Department of Nursing, The First Affiliated Hospital, Sun Yat-sen University, Guangzhou, China.; ^7^Australian National Institute of Management and Commerce, Sydney, NSW, Australia.; ^8^School of Economics and School of Management, Tianjin Normal University, Tianjin, China.; ^9^Guangdong Institute for International Strategies, Guangdong University of Foreign Studies, Guangzhou, China.; ^10^Newcastle Business School, University of Newcastle, Newcastle, NSW, Australia.; ^11^School of Management, University of Liverpool, Liverpool, UK.; ^12^China Center for Health Development Studies, Peking University, Beijing, China.

**Keywords:** Stroke, Comorbidity, Mortality, Costs, Length of Stay

## Abstract

**Background:** In this study, we examined the length of stay (LoS)-predictive comorbidities, hospital costs-predictive comorbidities, and mortality‐predictive comorbidities in immobile ischemic stroke (IS) patients; second, we used the Charlson Comorbidity Index (CCI) to assess the association between comorbidity and the LoS and hospitalization costs of stroke; third, we assessed the magnitude of excess IS mortality related to comorbidities.

**Methods:** Between November 2015 and July 2017, 5114 patients hospitalized for IS in 25 general hospitals from six provinces in eastern, western, and central China were evaluated. LoS was the period from the date of admission to the date of discharge or date of death. Costs were collected from the hospital information system (HIS) after the enrolled patients were discharged or died in hospital. The HIS belongs to the hospital’s financial system, which records all the expenses of the patient during the hospital stay. Cause of death was recorded in the HIS for 90 days after admission regardless of whether death occurred before or after discharge. Using the CCI, a comorbidity index was categorized as zero, one, two, and three or more CCI diseases. A generalized linear model with a gamma distribution and a log link was used to assess the association of LoS and hospital costs with the comorbidity index. Kaplan–Meier survival curves was used to examine overall survival rates.

**Results:** We found that 55.2% of IS patients had a comorbidity. Prevalence of peripheral vascular disease (21.7%) and diabetes without end-organ damage (18.8%) were the major comorbidities. A high CCI=3+ score was an effective predictor of a high risk of longer LoS and death compared with a low CCI score; and CCI=2 score and CCI=3+ score were efficient predictors of a high risk of elevated hospital costs. Specifically, the most notable LoS-specific comorbidities, and cost-specific comorbidities was dementia, while the most notable mortality-specific comorbidities was moderate or severe renal disease.

**Conclusion:** CCI has significant predictive value for clinical outcomes in IS. Due to population aging, the CCI should be used to identify, monitor and manage chronic comorbidities among immobile IS populations.

## Background

**Key Messages**

** Implications for policy makers**

- The Charlson Comorbidity Index (CCI) has significant predictive value for clinical outcomes in immobile patients with ischemic stroke (IS).
- Health insurance plans should use the CCI to assess and identifies the complexity of stroke-related diseases and the current disease status (such as hypertension, dementia, and diabetes) of the insured member.
- For clinical staff, CCI should be used to assess IS patients most likely to experience the worse clinical outcomes and assess, monitor and treat comorbidities during hospitalization.
- Due to population aging, the CCI should be used to identify, monitor and manage chronic comorbidities among the immobile IS population. We also recommend establishing a geriatric care system, where the CCI can be optimized in patient treatment.

** Implications for the public**

Stroke sufferers commonly have comorbidities, such as hypertension or diabetes, that directly or indirectly present barriers to optimal stroke recovery. The results indicate that comorbidities were central drivers of length of stay (LoS), hospital cost, and mortality in Chinese immobile ischemic stroke (IS) patients. Assessing the association between comorbidity and the LoS and hospitalization costs of stroke, and understanding the magnitude of excess IS mortality related to comorbidities, promises both to save the lives of stroke sufferers through integrating medical interventions according to their comorbidities and to better manage hospital costs of IS stroke patients with comorbidities.

Stroke is the second most common cause of death and disability worldwide.^1-3^ Globally, there were 80.1 million prevalent cases of stroke, with 84.4% ischemic stroke (IS).^4^ In 2016, 5.5 million died of stroke, and IS accounted for 49.1% of all stroke deaths.^4^ Stroke has been the leading cause of death in China in recent years,^5^ with 1.8 million annual deaths, accounting for roughly 30% of worldwide stroke mortality.^5-7^ Stroke also imposes a significant economic burden on society, families and individuals.^8^ In 2015, the annual cost for stroke care was estimated to RMB37.5 billion,^9^ and 2017 hospitalization cost per IS patient was RMB10 131, while China’s per capita disposable income was only RMB25 974.^10^ With increasing life expectancy and population aging, the stroke burden will pose an even more serious healthcare and economic challenge to China.^11^

 Stroke sufferers commonly have comorbidities, such as hypertension, cardiovascular disease, diabetes, or other ailments, including bleeding, medical complications, or stroke recurrence,^12-14^ that directly or indirectly present barriers to optimal stroke recovery.^15,16^ China has 7.5 million stroke survivors who suffered from comorbidities.^17^ These stroke survivors usually spend a significant period immobilized in bed,^2,18,19^ leading to personal functional limitations, loss of muscle mass, and medical complications.^20,21^ Comorbidity and immobility have a substantial effect on the final outcome of stroke patients, and often impede recovery, impacting directly or indirectly length of stay (LoS) in hospital, hospital costs, and death.^15,16^

 The impact of comorbidity on survival after hemorrhagic stroke among dialysis patients has been studied in Taiwan province.^22^ Yang et al examined the mortality-specific comorbidity among inpatients with IS in Sichuan province from 2012 to 2017.^23^ However, in China, there has been no nation-wide research on the LoS-specific comorbidities, hospital costs-specific comorbidities, and mortality‐specific comorbidities of immobile patients with IS. Assessing the association between comorbidity and the LoS and hospitalization costs of stroke, and understanding the magnitude of excess IS mortality related to comorbidities, promises both to save the lives of stroke sufferers through integrating medical interventions according to their comorbidities and to better manage hospital costs of IS stroke patients with comorbidities.^24^ This study examines the LoS-predictive comorbidities, hospital costs-predictive comorbidities, and mortality‐predictive comorbidities in immobile IS patients; second, we used the Charlson Comorbidity Index (CCI) to assess the association between comorbidity and the LoS and hospitalization costs of stroke; third, we assessed the magnitude of excess IS mortality related to comorbidities. We use a unique, large scale hospital-based multicenter dataset with an exhaustive discharge diagnosis to measure the stroke burden nationally of immobility IS patients with comorbidities.

## Materials and Methods

###  Study Design and Population

 Supported by National Health and Family Planning Commission’s agenda to improve the outcomes among immobile patients with stroke, the target population is all immobile stroke inpatients in 25 general hospitals in China. In order to ensure the representativeness of the study sample, between November 2015 and July 2017, we collected stroke patient data in 25 general hospitals in eastern China (Guangdong province, Zhejiang province, and Beijing municipal city), western China (Sichuan province), and central China (Henan province and Hubei province),^19^ comprising six tertiary hospitals, 11 secondary hospitals, and eight community hospitals. Study subjects met the following inclusion criteria: (*i*) aged 18 years or older; (*ii*) immobile, where the patient’s basic physiological needs were carried out in bed, except for active or passive bedside sitting/standing/wheelchair use for examination; (*iii*) ability to understand the study’s aims and to sign the consent form; (*iv*) diagnosed with IS as the major illness in their medical records. IS was defined according to the World Health Organization (WHO) definition as focal neurological deficit lasting for 24 hours with no apparent other than vascular cause,^25^ which was coded I63.x in the International Classification of Disease, 10th revision (ICD-10) based on pathological subtypes.^2,26,27^ All eligible inpatients of the selected hospitals were continuously enrolled. A total of 5114 participants enrolled in the study, with follow-ups continuing 90 days after enrollment unless they died in hospital or relinquished medical treatment. Survival time was measured by the duration from the date of hospital admission to the date of death and LoS was defined as the period from the date of admission to the date of hospital discharge or date of death, which is an important performance indicator of hospital management.^28-30^ Hospital costs were derived from the hospital information system (HIS) in each hospital after the enrolled patients died or were discharged from hospital. The HIS belongs to the hospital’s financial system, which records all the expenses of the patient during the hospital stay. The listed cause of death of the IS patient was recorded in the HIS for 90 days after the date of admission regardless of whether death occurred before or after discharge. Dates of death were collected from standardized HIS case report forms.

###  Definition of CCI and Covariates

 Comorbidity was assessed using the CCI, which identifies multiple comorbidities. Adjusted specifically for stroke evaluation,^31-33^ the CCI accounts for multiple comorbidities by creating a sum score, weighted according to the presence of 19 comorbid conditions.^16,26,34^ We used the actual weighted score derived from the CCI. Different comorbid conditions had different weights in CCI. For example, Table 1 shows that myocardial infarction is weighted 1; diabetes with end-organ damage is weighted 2; and moderate or severe liver disease is weighted 3.^23,26,34,35^ CCI was designed to enable researchers to control for the prognostic impact of other chronic diseases on the outcomes of patients with a specific disease, and it is commonly used in outcome and mortality studies.^15,34,36^ The CCI data in our study were derived from the discharge ICD-10 codes and patient histories obtained from the HIS standardized case report forms. Following previous studies, the total CCI score for each patient was categorized into four levels of comorbidity, 0 (none), 1 (moderate), 2 (severe), and 3+ (very severe).

**Table 1:** Sociodemographic and Clinical Characteristics of the Patients (N* = *5114)

**Variable**	**Number (%)**
Gender	
Male	3028 (59.2)
Female	2086 (40.8)
Age	
0-59	1278 (25.0)
60-69	1348 (26.4)
70-79	1340 (26.2)
80+	1148 (22.4)
Education	
Illiteracy	1037 (20.3)
Primary school	1781 (34.8)
Junior high school	1226 (24.0)
High school and above	1070 (20.9)
Insurance	
No insurance^a^	815 (15.9)
NCMS	2070 (40.5)
URBMI	981 (19.2)
UEBMI	1248 (24.4)
CCI score	
0	2291 (44.8)
1	1600 (31.3)
2	704 (13.8)
3+	519 (10.1)
Individual CCI diseases (Ref = Without comorbidity)	
Myocardial infarction (weight = 1)	63 (1.2)
Congestive heart failure (weight = 1)	576 (11.3)
Peripheral vascular disease (weight = 1)	1108 (21.7)
Dementia (weight = 1)	153 (3.0)
Chronic pulmonary disease (weight = 1)	468 (9.2)
Connective tissue disease (weight = 1)	61 (1.2)
Ulcer disease (weight = 1)	49 (1.0)
Mild liver disease (weight = 1)	304 (5.9)
Diabetes without end-organ damage (weight = 1)	960 (18.8)
Hemiplegia (weight = 2)	15 (0.3)
Moderate or severe renal disease (weight = 2)	199 (3.9)
Diabetes with end-organ damage (weight = 2)	106 (2.1)
Tumor without metastasis (weight = 2)	150 (2.9)
Leukemia (weight = 2)	2 (0.0)
Lymphoma (weight = 2)	6 (0.1)
Moderate or severe liver disease (weight = 3)	53 (1.0)
Metastatic solid tumor (weight = 6)	41 (0.8)
AIDS (weight = 6)	3 (0.1)
Total	5114 (100)

Abbreviations: CCI, Charlson Comorbidity Index; NCMS, New Cooperative Medical System (covered rural residents); URBMI, Urban Resident Basic Medical Insurance (covered urban residents without a stable job); UEBMI, Urban Employee Basic Medical Insurance (covered employed workers); AIDS, acquired immune deficiency syndrome. ^a^No insurance (patients pay all hospital fees out of pocket).

 We identified all 19 comorbidities included in the CCI,^15,36,37^ except for cerebrovascular disease. Control variables included gender (male or female), age (0-59, 60-69, 70-79, 80 years or older), education level (illiteracy, primary school, junior high school, high school and above), and type of insurance.^38^ The type of insurance comprised no insurance, New Cooperative Medical System (NCMS) for rural residents,^39^ Urban Resident Basic Medical Insurance (URBMI) for urban unemployed, elderly, children and students,^40^ and Urban Employee Basic Medical Insurance (UEBMI) for urban employed and retired.^41^ NCMS, UEBMI and URBMI were China’s three basic social health insurance schemes, but differed in reimbursement ratios, coverage and contributions.^38^ Previous research indicated that medical insurance was associated with the cost and adverse clinical outcomes of stroke,^10,42-44^ therefore, we included the type of insurance in the analysis.

###  Statistical Analyses

 We employed descriptive statistics to summarize patients’ characteristics. In the univariate analysis, LoS and hospital costs were compared between different subgroups using the rank-sum test, and death proportions were compared between different subgroup using the χ^2^ test. Generalized linear model with a gamma distribution and a log link was used to assess the association of LoS and hospital costs with the CCI score. The results were reported as percentage changes (= exp^^coefficient^-1) and 95% confidence intervals (CIs) in LoS and hospital costs for IS, in association with the presence of comorbidities. Kaplan–Meier survival curves was used to examine overall survival. Using multivariable Cox regression models, adjusted for gender, age, education level, and type of insurance, the relative risk of death of all mortality causes was expressed as hazard ratios (HRs) with 95% CIs. In order to further analyze the specific types of comorbidity, we identified the nine most prevalent types of comorbidities as binary variables: peripheral vascular disease, diabetes without end-organ damage, congestive heart failure, chronic pulmonary disease, mild liver disease, moderate or severe renal disease, dementia, tumor without metastasis, and diabetes with end-organ damage. A *P *value of less than.05 was considered statistically significant. Statistical analysis was performed with Stata version 14 for Windows (Stata Corp, College Station, TX, USA).

## Results

###  Demographic Information of Stroke Patients

 Descriptive statistics are shown in Table 1 and Table 2. Among the 5114 participants, 3028 (59.2%) were male; there is no statistical difference between males and females in terms of the LoS (18 days – standard deviation [SD] ± 15.4) versus 17 days (SD ±13.2; *P* = .069) and death (7.4% versus. 6.5%; *P* = .211), while there is a significance difference between the average hospital costs of males and females(RMB40 047 [SD ±RMB57 705.5] versus RMB35 339 [SD ±RMB48152.9]; *P* < .001). Three quarters of the participants were older than 60 years, and 22.4% older than 80 years. Participants younger than 59 years had higher average hospital costs than the elderly, while increasing age increased the risk of mortality. More than one third of participants had a primary school background and 20.9% had a high school and above education level. With regard to insurance type, 2070 (40.5%) participants were covered by NCMS, 24.4% by UEBMI and 19.2% by URBMI, and 15.9% had no insurance. The proportion of participants with no comorbidities was 44.8%, with 31.3% with one, 13.8% with two and 10.1% with three or more comorbidities. The most frequent comorbid conditions in Table 1 were peripheral vascular disease (21.7%), diabetes without end-organ damage (18.8%), congestive heart failure (11.3%), chronic pulmonary disease (9.2%), and mild liver disease (5.9%). There is a significance difference between the different CCI scores in terms of the LoS, costs, and death (*P* < .001). The data for hospitalizations, analyzed according to CCI scores, are presented in Table 2.

**Table 2:** Data for Hospitalizations, Analyzed According to the CCI Score (N* = *5114)

	**Average LoS (days)**		**Average Hospital Cost (RMB)**		**Death**	
	**Mean (SD)**	***P*** ** Value**	**Mean (SD)**	***P*** ** Value**	**No. (%)**	***P*** ** Value**
CCI score		<.001		<.001		<.001
0	17 (13.2)		39 407 (53 678.6)		131 (5.7)	
1	16 (13.8)		32 686 (48 474.1)		78 (4.9)	
2	18 (13.2)		38 714 (51 411.7)		67 (9.5)	
3+	21 (22.0)		48 329 (71 150.2)		85 (16.4)	
Individual CCI diseases (Ref = Without comorbidity)						
Myocardial infarction (weight = 1)	20 (26.4)	.631	66 871 (67 585.0)	<.001	14 (22.2)	<.001
Congestive heart failure (weight = 1)	20 (20.5)	.003	43 334 (55 864.8)	.025	76 (13.2)	<.001
Peripheral vascular disease (weight = 1)	17 (15.7)	.785	30 228 (47 272.7)	<.001	51 (4.6)	<.001
Dementia (weight = 1)	28 (35.5)	.082	48 100 (92 767.8)	.525	19 (12.4)	.076
Chronic pulmonary disease (weight = 1)	20 (19.6)	<.001	38 941 (64 630.4)	.073	45 (9.6)	.009
Connective tissue disease (weight = 1)	16 (10.5)	.035	49 551 (67 757.4)	.265	7 (11.5)	.024
Ulcer disease (weight = 1)	18 (13.5)	.865	43 471 (52 385.3)	.025	4 (8.2)	.175
Mild liver disease (weight = 1)	16 (10.8)	.634	35 491 (44 789.1)	.163	8 (2.6)	.762
Diabetes without end-organ damage (weight = 1)	18 (15.4)	.110	34 663 (54 450.3)	.590	72 (7.5)	.002
Hemiplegia (weight = 2)	36 (21.1)	.106	57 205 (65 431.0)	.032	0 (0.0)	.554
Moderate or severe renal disease (weight = 2)	19 (17.7)	<.001	49 081 (72 738.2)	.066	51 (25.6)	.285
Diabetes with end-organ damage (weight = 2)	19 (16.4)	.055	39 260 (43 334.4)	.008	12 (11.3)	.000
Tumor without metastasis (weight = 2)	19 (11.2)	.858	60 971 (59 852.1)	.207	32 (21.3)	.083
Leukemia (weight = 2)	8 (3.5)	<.001	68 841 (64 074.4)	<.001	2 (100.0)	<.001
Lymphoma (weight = 2)	46 (51.3)	.088	102 382 (104 227.9)	.240	1 (16.7)	<.001
Moderate or severe liver disease (weight = 3)	20 (18.2)	.062	67 425 (75 159.7)	.005	10 (18.9)	.358
Metastatic solid tumor (weight = 6)	16 (8.3)	.684	51 599 (33 116.5)	<.001	14 (34.1)	.001
AIDS (weight = 6)	9 (3.5)	.407	19 230 (6483.2)	<.001	0 (0.0)	<.001
Total	17.1 (14.6)	.082	38 135.7 (54 075.7)	.832	361.0 (100.0)	.633

Abbreviations: LoS, length of stay; CCI, Charlson Comorbidity Index; SD, standard deviation; RMB, renminbi.

###  Length of Stay

**Figure 1:**
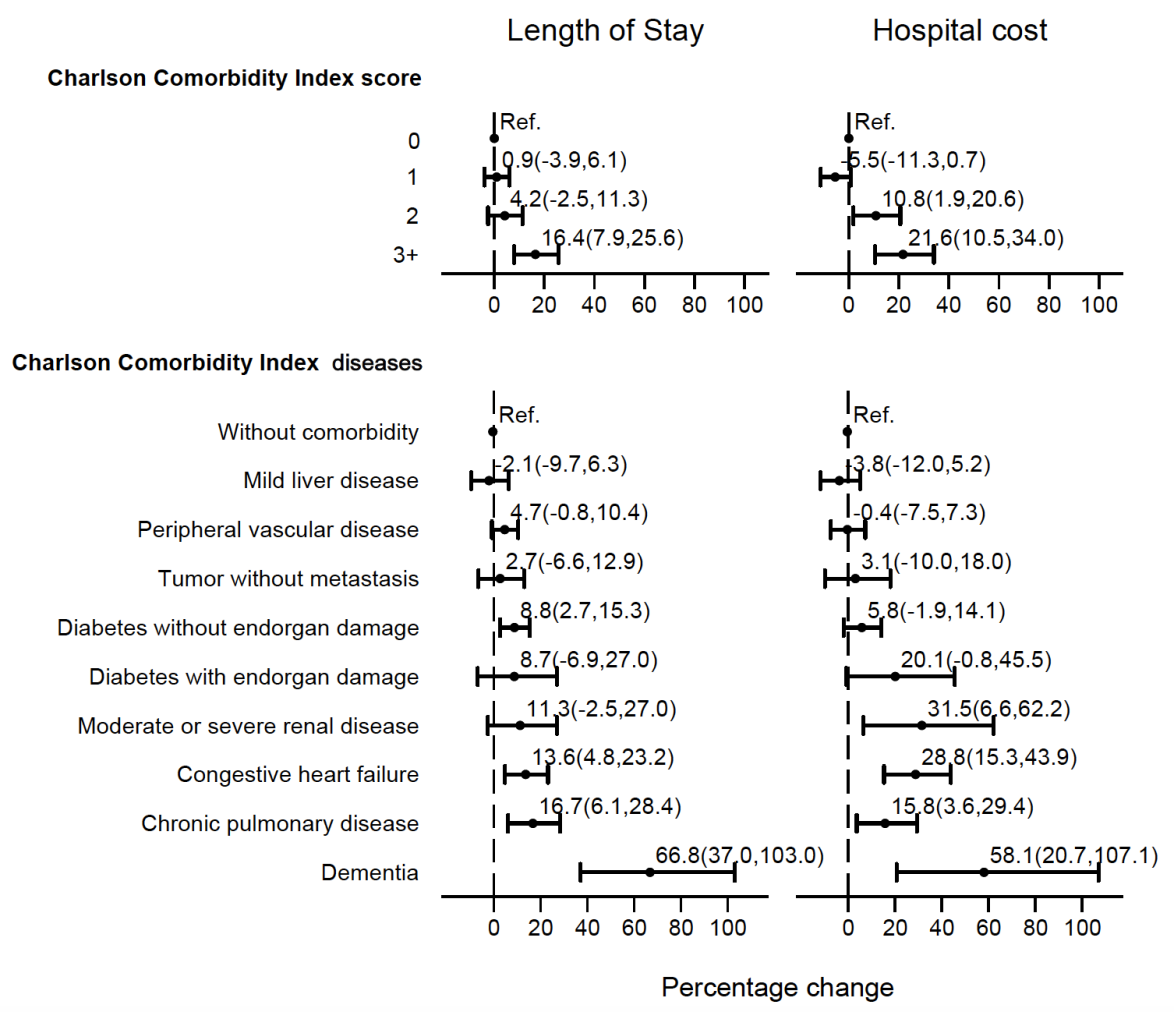
Percentage Change and 95% CI in LoS and Hospital Cost for IS Associated With Comorbid Conditions Adjusted for Gender, Age, Year of Diagnosis, Education Level, and Type of Insurance. Abbreviations: LoS, length of stay; SD, standard deviation; IS, ischemic stroke; CI, confidence interval.

Table 2 also displays the LoS among the study participants, with average LoS in the CCI = 0 group 17 days (SD ±13.2); CCI = 1 group 16 days (SD ±13.8), CCI = 2 group 18 days (SD ±13.2) and CCI = 3+ group 21 days (SD ±22.0). Figure 1 depicts the percentage change and 95% confidence interval (CI) in LoS associated with comorbidities. After adjusting for potential confounders (Table S1, see Supplementary file 1), compared with CCI = 0 score, there was no significance differences in CCI = 1 (0.9%, 95% CI -0.039 to 0.061; *P* = .712) and CCI = 2 (4.2%, 95% CI -0.025 to 0.113; *P* = .225), whereas a high CCI = 3+ score was associated with a significantly 16.4% higher likelihood of increased LoS compared with a CCI = 0 score (16.4%, 95% CI 0.079 to 0.256; *P* < .001).

 Compared with the no comorbid condition, the percentage change in LoS was significant among IS patients with dementia (66.8%, 95% CI 0.370 to 1.030; *P* < .001), chronic pulmonary disease (16.7%, 95% CI 0.061 to 0.284;* P* = .002), congestive heart failure (13.6%, 95% CI 0.048 to 0.232;* P* = .002), and diabetes without end-organ damage (8.8%, 95% CI 0.027 to 0.153;* P* = .004).

###  Hospital Costs

Table 2 also shows average hospitalization costs, with the mean costs in the CCI = 0 group RMB39 407 (SD ±RMB53 678.6); CCI = 1 group RMB32 686 (SD ±RMB4 8474.1); CCI = 2 group RMB38 714 (SD ±RMB51 411.7); and CCI = 3+ group RMB48 329 (SD ±RMB71 150.2). Figure 1 presents the percentage change and 95% CI in hospital cost associated with comorbid conditions. As shown in Table S1, after adjusting for control variables, the CCI = 1 group (-0.055, 95% CI -0.113 to 0.007; *P* = .083) was not significant, but the CCI = 2 group (0.108, 95% CI 0.019 to 0.206; *P* = .017) was associated with a 10.8%, and CCI = 3+ group (0.216, 95% CI 0.105 to 0.340; *P* < .001) a 21.6%, higher likelihood of increased hospital costs compared with a CCI = 0 scores. Compared with no comorbidity, the percentage change was most notable among IS patients with dementia (58.1%, 95% CI 0.207 to 1.071;* P* < .001), moderate or severe renal disease (31.5%, 95% CI 0.066 to 0.622;* P* = .011), congestive heart failure (28.8%, 95% CI 0.153 to 0.439;* P* < .001), and chronic pulmonary disease (15.8%, 95% CI 0.036 to 0.294;* P* = .009).

###  Mortality

**Figure 2:**
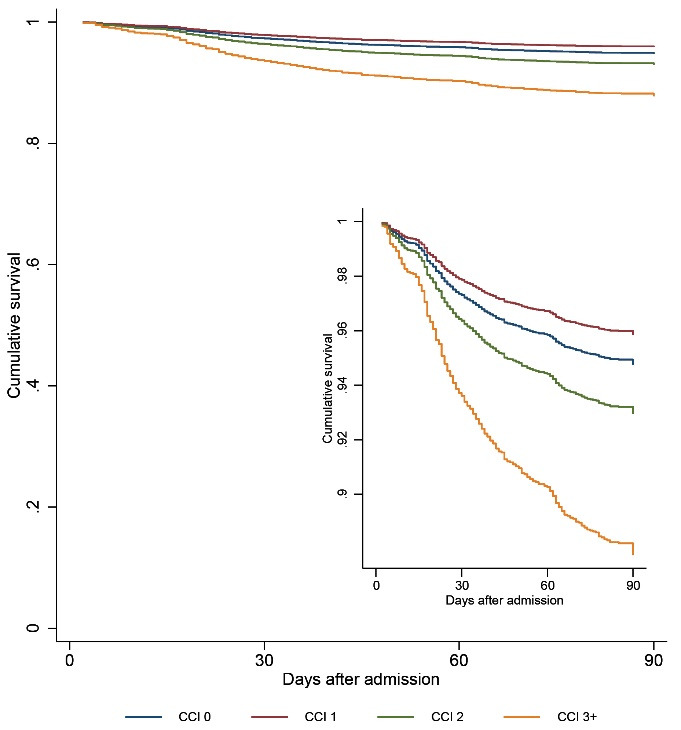
Cumulative Survival by CCI Score and Adjusted for Gender, Age, Education Level, and Type of Insurance. The smaller figure within Figure 2, adjusted the starting point of the vertical axis to present the differences between the groups more prominently. Abbreviation: CCI, Charlson Comorbidity Index.

 The overall death rate was 7.06%, with the CCI = 0 group accounting for 5.7% of deaths, the CCI = 1 group accounting for 4.9% of deaths, CCI = 2 group accounting for 9.5% of deaths and CCI = 3+ accounting for 16.4% of deaths. Figure 2 shows that the cumulative survival by CCI group, with patients with high comorbidity (CCI = 3+) having significantly poorer survival rates than lower CCI groups. As shown in Table 3, compared with CCI = 0 group, there was no significant difference between the CCI = 1 group (HR 0.789, 95% CI 0.590 to 1.055; *P* = .110) and CCI = 2 group (HR 1.356, 95% CI 0.989 to 1.859; *P* = .059) in the Cox regression model after adjusting for confounders. However, as shown in Table 3, a high CCI = 3+ score was positively correlated with approximately a 2.5-fold higher risk of death compared to a CCI = 0 score group after adjusting for confounders (HR 2.419, 95% CI 1.808 to 3.235; *P* < .001).

**Table 3:** Results From Cox Regression Model

**Variable **	**HR (95% CI)**
CCI score (Ref. = 0)	
1	0.789 (0.590-1.055)
2	1.356 (0.989-1.859)
3+	2.419 (1.808-3.235)^a^
Individual CCI diseases (Ref. = Without comorbidity)	
Peripheral vascular disease	0.656 (0.466-0.925)^a^
Diabetes without end-organ damage	1.282 (0.947-1.734)
Congestive heart failure	1.837 (1.341-2.517)^b^
Chronic pulmonary disease	1.221 (0.843-1.768)
Mild liver disease	0.521 (0.254-1.069)
Moderate or severe renal disease	3.992 (2.795-5.702)^a^
Dementia	1.488 (0.871-2.543)
Tumor without metastasis	3.361 (2.214-5.101)^a^
Diabetes with end-organ damage	1.986 (1.061-3.716)^b^

Abbreviations: CCI, Charlson Comorbidity Index; HR, hazard ratio; CI, confidence interval. All models adjusted for gender, age, year of diagnosis, education level, and type of insurance. ^a^*P* <.001, ^b^*P* <.05.

 As shown in Table 3, the relative risk of death from all causes were most notable among IS with moderate or severe renal disease (HR 3.992, 95% CI 2.795 to 5.702; *P* < .001), followed by patients with tumor without metastasis (HR 3.361, 95% CI 2.214 to 5.101; *P* < .001), congestive heart failure (HR 1.837, 95% CI 1.341 to 2.517; *P* < .001), diabetes with end-organ damage (HR 1.986, 95% CI 1.061 to 3.716; *P* = .032), and peripheral vascular disease (HR 0.656, 95% CI 0.466 to 0.925; *P* = .016).

## Discussion

 Our study systematically examined the associations between comorbidity and LoS, hospital costs, and mortality among immobile IS patients. Overall, 55.2% of our sample’s IS patients had a comorbid condition, with a high CCI score associated with increased average LoS, higher hospital costs, and a greater likelihood of death.

 The prevalence estimates of comorbidities among immobile IS patients in China was 55.2%, which is lower than a retrospective analysis extracted from the Sichuan Provincial People’s Hospital database reporting that 79% of inpatients with IS had at least one comorbidity.^23^ Previous research indicated that 94.2% of stroke sufferers have one or more comorbid conditions in Scotland,^45^ and the prevalence of comorbidity ranged from 44% in Denmark^34^ to 99% in Ontario, Canada.^46^ These differences are likely due to a variety of factors, including the study design,^23^ study periods,^34^ study subjects,^46^ local factors (such as the standard of nursing care or medical treatment received),^33^ and the use of different classifications or ICD codes for determining comorbidities.^16^

 We found the most frequent comorbid condition was peripheral vascular disease (21%). There are a number of possible explanations for this situation. Participants enrolled in our study were immobile, and the literature indicates that peripheral vascular disease are closely related to immobilization.^14,47^ Second, there are some common potential risk factors for both peripheral vascular disease and IS, including hypertension, alcohol assumption and atherosclerosis.^48-51^ We cannot, however, infer the sequence and the causality of IS and peripheral vascular disease. Since vascular examinations were routinely conducted for immobile patients (especially for those with suspected cardiovascular and cerebrovascular diseases), tests such as Doppler ultrasound, computed tomography angiography, magnetic resonance angiography, may contribute to the higher prevalence of peripheral vascular disease in our study.^23^ This result suggests that early tests, such as Doppler ultrasound, may improve the detection rate of peripheral vascular disease in immobile IS patients, and prospective clinical study is required to justify this issue.

 Our results suggested that a CCI = 2 score was related to higher hospital costs, and a high CCI = 3+ score was positively correlated with both longer LoS and higher hospital costs, which supports CCI as a measure for the disease burden of IS.^26,33^ Since CCI = 3+ group had a higher likelihood of increased LoS and hospital costs compared with a CCI = 0 score, with similar results for the CCI = 1 group and CCI = 2 group, CCI = 3+ patients need more attention during hospitalization. Dementia, chronic pulmonary disease, and congestive heart failure were LoS and hospital cost‐specific comorbidities. One explanation is that approximately three in four participants enrolled in our study were the elderly, and these chronic diseases tend to cluster in cohorts with increasing age.

 We also found that high comorbidity as measured by the CCI = 3+ was associated with an approximately 2.5-fold higher risk of death than a low CCI = 0 score. This confirms the use of CCI to predict short-term death in the Chinese IS population is meaningful. Our results also indicate that moderate or severe renal disease and tumor without metastasis increased the death rate. This can be explained by stroke rates for end-stage renal disease patients and cancer are substantially higher than for the general population, and those renal and cancer patients have a poor prognosis and high mortality rate, despite advances in diagnosis and treatment.^15,52^

 The association between the scores of CCI and multiple clinical outcomes, including LoS, hospital costs, and mortality, supports the notion that the CCI should be used to identify and manage chronic comorbidities,^16,22,33,36^ which identify patients with multiple chronic diseases in a universally applicable, transparent, and auditable method, through a weighted measure of the burden of chronic disease that predicts long term prognosis, outcomes and costs.^53,54^ In addition, the CCI was designed to enable researchers to control for the prognostic impact of other chronic diseases on the health outcomes of patients with a specific chronic disease. Without CCI, researchers often excluded patients with other chronic diseases from their studies to eliminate the potential of ‘confounding’ factors. For instance, studies of patients with diabetes might exclude patients with dementia or cancer to make certain that they did not confound the clinical outcomes. Such exclusions limited the number of participants to whom the study results applied.^31,34^ Further, reference to a CCI indicator is both a simple and more direct method than a nonstandard list of a variety of different chronic disease conditions, which facilitates doctors’ decision-making. Therefore, knowing a patient has a CCI score of 3+ provides more useful information than just knowing if the patient has hypertension or dementia. In the future, health insurance plans should require and use the CCI to identify the complexity of stroke-related diseases and the current chronic disease status (such as hypertension, dementia and diabetes) of the insured member. Based on the stratification of zero, one, two, and three or more CCI diseases, and the patients’ comorbid conditions, the government should increase the financial investment, raise reimbursement rates and set up differential reimbursements to meet the health needs of patients with multiple diseases. For clinical staff, CCI should be used to assess, monitor, and treat comorbidities during hospitalization.^55^ The early use of the CCI plays a critical role in the identification of IS patients most likely to experience the worse clinical outcomes and may help target interventions according to the resulting risk information, which can tackle such conditions and reduce their pernicious effects.

 China is encountering formidable healthcare challenges related to population aging, with 12% of the population over 65 years old in 2020, which is forecast to rise to 26% by 2050.^56^ Comorbidities associated with the aging population will pose a significant challenge to China’s health system. Combined with tailored stroke interventions according to patient comorbidity, we recommend the establishment of a geriatric care system to improve the health of the stroke-prone population and reduce the stroke burden on the health system.

 Our study has some limitations. Common to health research, a retrospective post-hoc analysis study design was used in the current study, where existing data was analyzed to answer new questions. For example, some clinical data, such as the details of the cause of death, laboratory data, clinical severity of stroke and premorbid Rankin scale data were not recorded in the HIS, which is an inherent drawback in retrospective analysis of a prospectively collected database. Second, no data on the severity of comorbidities was available. Third, we cannot infer the sequence and the causality of IS and peripheral vascular disease. Fourth, diagnostic validity of comorbidity might be influenced by heterogeneity of physician documentation and code assignment accuracy from different sites. Prospective studies with more sophisticated evaluations are required to address these limitations.

## Conclusion

 Comorbidities were central drivers of LoS, hospital cost, and mortality in Chinese immobile IS patients. We found CCI had a significant predictive value for clinical and economic outcomes in IS patients. Due to population aging, the CCI should be used to identify, monitor and manage chronic comorbidities among the immobile IS population. We also recommend establishing a geriatric care system, where the CCI can be optimized in patient treatment.

## Acknowledgements

 The authors thank research participants and nursing staff for their kind and efficient contribution to the study. We acknowledge the helpful comments by three anonymous reviewers.

## Ethical issues

 The Ethics Committee of Peking Union Medical College Hospital (S-700) approved the project. All patients provided written informed consent before enrollment in the study, and all IS patients coded I63.x according to the ICD-10 enrolled in the current study (N = 5114). Patients’ records and information were anonymized and de-identified prior to the analysis.

## Competing interests

 Authors declare that they have no competing interests.

## Authors’ contributions

 Study concept and design: XW. Analysis and interpretation of data: HL and DZ. Editing of the manuscript and drafting of tables: HL and DZ. Critical review of the manuscript for important intellectual content: XW, HL, and DZ. Patient recruitment, data collection, and manuscript editing: BS, JJ, YL, XW, SC, EM and SN. All authors critically reviewed and approved the manuscript before it was submitted.

## Supplementary files

Supplementary file 1 contains Table S1.
