# Supplementary material for: Length of Stay, Hospital Costs and Mortality Associated With Comorbidity According to the Charlson Comorbidity Index in Immobile Patients After Ischemic Stroke in China: A National Study
Source: Int J Health Policy Manag. 2021 Aug 7;11(9):1780–7. doi: 10.34172/ijhpm.2021.79 (PMC9808248; doi:10.34172/ijhpm.2021.79)
Supplement: Supplementary file 1 — contains Table S1. [file ijhpm-11-1780-s001.pdf]

Article title: Length of Stay, Hospital Costs and Mortality Associated with Comorbidity According to the Charlson Comorbidity Index in Immobile Patients After Ischemic Stroke in China: A National Study

Journal name: International Journal of Health Policy and Management (IJHPM)

Authors' information: Hongpeng Liu[1], Baoyun Song[2], Jingfen Jin[3], Yilan Liu[4], Xianxiu Wen[5], Shouzhen Cheng[6], Stephen Nicholas[7,8,9,10], Elizabeth Maitland[11], Xinjuan Wu[1]*, Dawei Zhu[12]*

[1]Department of Nursing, Chinese Academy of Medical Sciences – Peking Union Medical College, Peking Union Medical College Hospital, Beijing, China.

[2]Department of Nursing, Henan Provincial People's Hospital, Zhengzhou, China.

[3]Department of Nursing, The Second Affiliated Hospital Zhejiang University School of Medicine, Hangzhou, China.

[4]Department of Nursing, Wuhan Union Hospital, Wuhan, China.

[5]Department of Nursing, Sichuan Provincial People's Hospital, Chengdu, China.

[6]Department of Nursing, The First Affiliated Hospital, Sun Yat-sen University, Guangzhou, China.

[7]Australian National Institute of Management and Commerce, Sydney, NSW, Australia.

[8]School of Economics and School of Management, Tianjin Normal University, Tianjin, China.

[9]Guangdong Institute for International Strategies, Guangdong University of Foreign Studies, Guangzhou, China.

[10]Newcastle Business School, University of Newcastle, Newcastle, NSW, Australia.

[11]School of Management, University of Liverpool, Liverpool, UK.

[12]China Center for Health Development Studies, Peking University, Beijing, China.

(*corresponding author: Email: wuxinjuan@sina.com & zhu_dawei@163.com)

Supplementary file 1

Table S1. Adjusted Association Between Comorbid Conditions With Length of Stay and Hospital Cost

	Length of stay		Hospital cost	
	Coefficient (95%CI)	*P* value	Coefficient (95%CI)	*P* value
CCI score (Ref.=0)				
1	0.009(-0.039-0.061)	0.712	-0.055(-0.113-0.007)	0.083
2	0.042(-0.025-0.113)	0.225	0.108(0.019-0.206)	0.017
3+	0.164(0.079-0.256)	<0.001	0.216(0.105-0.340)	<0.001
Individual CCI diseases (Ref.=Without comorbidity)				
Peripheral vascular disease	0.047(-0.008-0.104)	0.098	-0.004(-0.075, 0.073)	0.925
Diabetes without end-organ damage	0.088(0.027-0.153)	0.004	0.058(-0.019-0.141)	0.143
Congestive heart failure	0.136(0.048-0.232)	0.002	0.288(0.153-0.439)	<0.001
Chronic pulmonary disease	0.167(0.061-0.284)	0.002	0.158(0.036-0.294)	0.009
Mild liver disease	-0.021(-0.097-0.063)	0.619	-0.038(-0.120, 0.051)	0.397
Moderate or severe renal disease	0.113(-0.025-0.270)	0.114	0.315(0.066-0.622)	0.011
Dementia	0.668(0.370-1.030)	0.000	0.581(0.207-1.071)	0.001
Tumor without metastasis	0.027(-0.066-0.129)	0.581	0.031(-0.100, 0.180)	0.661
Diabetes with end-organ damage	0.087(-0.069-0.270)	0.290	0.201(-0.008, 0.455)	0.061

Abbreviation, CCI, Charlson Comorbidity Index.
